# A mixed methods survey of research education requirements for residents in internal medicine, neurology and transitional programs

**DOI:** 10.1080/10872981.2025.2494579

**Published:** 2025-04-24

**Authors:** Michael O’Shea, Nikita Ashcherkin, Suganya Arunachalam Karikalan, Matt Biondi, Hally Chaffin, Sarah Umar, Nathan Delafield, Nikita Chhabra, Matthew Hoerth, Amaal Starling, Umesh Sharma, Brittane Valles, Christina Wu, Gretchen Taylor, Camille Hawkins, Patress Persons

**Affiliations:** a Department of Internal Medicine, Mayo Clinic Arizona, Phoenix, AZ, USA; b Department of Internal Medicine, Christus Good Shepherd Medical Center, Longview, TX, USA; c Department of Gastroenterology, Mayo Clinic Arizona, Phoenix, AZ, USA; d Department of Neurology, Mayo Clinic Arizona, Phoenix, AZ, USA; e Department of Hematology and Oncology, Mayo Clinic Arizona, Phoenix, AZ, USA

**Keywords:** Curriculum design, research education, residency, medical education, neurology education

## Abstract

**Introduction:**

Participation in scholarly activity is an essential component and outcome metric of clinical training. Residency research education curricula have been independently developed by many institutions, however results from these programs vary.

**Methods:**

We conducted a survey of Internal Medicine, Neurology and Transitional Residents to determine knowledge, attitudes, beliefs, and practices. Likert-style, open and closed questions were used. Results were analyzed using preference ranking, thematic analysis, descriptive statistics, and chi squared and fisher’s exact test for association between categorical variables.

**Results:**

Prior exposure to formal research opportunities in medical school significantly correlated with publication rates (OR 2.37, *p* = 0.022) but did not predict continued engagement in research during residency. Residents expressed confidence in critical appraisal skills but reported low confidence in statistical analysis and study design. Observational study designs, particularly chart review cohort studies, were ranked as the most desirable research focus areas, whereas outpatient and community-based research were of lower priority. Barriers to research productivity included time constraints, statistical analysis challenges, methodological concerns, and lack of mentorship.

**Discussion:**

The findings underscore the need for structured approaches tailored to resident preferences to enhance scholarly engagement. Residents ranked observational study design and systematic reviews as their top priorities, indicating a preference for research methodologies that are practicable within residency timelines. Residency programs should enhance early mentorship, provide targeted research education, and facilitate access to statistical and methodological support to improve research productivity among trainees.

## Introduction

Active participation in scholarly activities is essential to the future development of clinician scientists, who will be uniquely positioned to address patient needs by conducting translational research. Objectively, the invaluable contribution of Medical Doctors to the collective knowledge base is reflected in key metrics, such as Nobel Prize Winners and patent applications [1]. Scholarly research activities, including research engagement, are an essential component of accredited residency programs and are monitored by the American College of Graduate Medical Education (ACGME) [2]. An individual’s research output during early clinical training can significantly determine access to future training opportunities such as sub-specialty fellowship training and career development grants [3].

High performing residency programs typically incorporate research tracks, have designated research directors, provide statistical support, and offer resident-specific funding [4]. Availability of formal research tracks and curricula can significantly improve research output among residents; however the content of these existing curricula differ substantially between programs and specialties, and impact on trainee research productivity is inconsistent [5–9]. Curricula often do not address perceived barriers to resident scientific productivity such as grant writing, regulatory issues and data analysis [4,10]. Existing survey data demonstrates that program directors may overestimate the abilities of trainees in these key areas and underestimate the extent to which their junior colleagues lack confidence in fundamental research tasks [11].

Research engagement during residency is influenced by structured programs, mentorship, and institutional support. High-functioning internal medicine residency programs highlight the role of research directors, formal curricula, and protecting time in fostering resident scholarship, while mentorship quality is a key determinant of research success [4,5]. Similarly, a structured protocol in cardiothoracic surgery significantly improved resident academic productivity by integrating dedicated mentorship, statistical support, and systematic project management [9]. Anesthesiology residency programs with formal research education curricula also demonstrated greater research output, with structured programs being associated with higher publication rates [6]. These findings underscore the importance of formalized research tracks and targeted support mechanisms in enhancing resident research productivity across specialties. However, little is known about resident preferences for research education curricula.

### Aims

This study aims to enable representation of trainee priorities in research curriculum development through a mixed methods knowledge, attitudes, beliefs, and practices (KABP) survey of transitional, internal medicine and neurology residents.

## Methods

### Survey design & pilot

We conducted a mixed method survey of Internal Medicine, Neurology and Transitional Residents in a single quaternary referral center. The survey was developed for the purpose of this study and piloted on a separate cohort of residents and fellows in other specialties for ease of use and validity using qualitative pre- and post-survey one-on-one interviews. Feedback from pilot testing prompted small modifications to question phrasing and format, enhancing clarity and alignment with the study’s objectives. Mixed method styles included Likert-style questions, multiple choice questions, rank based questions and open-ended questions (supplemental appendix 1).

### Conduct

The survey was circulated using Microsoft Forms via an institutional approved email template. Group consent to participate was obtained from Residency Program Directors and Assistant Program Directors, and an individual consent form was included in survey responses. The survey was circulated between 1 October 2024 and 2/16/2024.

### Analysis

Likert-style and rank questions were assessed using descriptive statistics and ranked according to mean preference. Association between responses and demographic and baseline characteristics was conducted using chi-squared test and fisher exact test. Fisher’s exact test was chosen to accommodate small sample sizes and to guarantee precise analysis of categorical variables. Association between ordered categorical responses was assessed using univariable logistic regression. Open ended questions were analyzed using thematic analysis with manual coding. Analysis was conducted using Stata IC version 16.

### Ethics

Approval was obtained for this study from the Mayo Clinic Institutional Review Board (ID 23–011040) and the Mayo Clinic Educational Research Committee (Application 23–107). An independent data administrator (SAK) not involved in any of the participating programs or departments was used to reduce the risk of coercion to participate and to protect participant anonymity.

## Results

49 of 67 (73.13%) residents completed the survey. 29 (59.18%) were Internal Medicine Residents, 10 were Neurology Residents (20.41%) and 10 (20.41%) were Transitional Year Residents.

### Determinants of resident success

“Formal research experiences during medical school were associated with higher odds of publication (OR 2.37 [1.13 to 4.96], *p* = 0.022) (Table 1). These medical school research opportunities did not translate into higher participation in research during residency (OR 1.35 [0.53 to 3.46], *p* = 0.528). This suggests that, while such opportunities are likely to result in publication, they do not necessarily translate into later research engagement. Formal teaching in clinical research methods was not associated with higher odds of publication (OR = 1, *p* = 1.00) or of engagement in clinical research during residency (OR = 1, *p* = 1.00). This suggests the training received in medical school is not sufficient in empowering residents to engage in productive research later on.

**Table 1.** Association between research outcomes and resident characteristics and experiences. P-values are calculated using chi-squared test for heterogeneity.

		n (%)	First author original research paper		Oral presentation		Poster presentation		Participating in research in residency	
			27 (56.25%)		11 (22.92%)		33 (70.21%)		39 (81.25%)	
			**OR (95% CI)**	**p-value**	**OR (95% CI)**	**P-value**	**OR (95% CI)**	**p-value**	**OR (95% CI)**	**p-value**
Female sex		19 (41.3%)	0.89 (0.27 to 2.93)	0.846	0.66 (0.14 to 3.10)	0.592	0.55 (0.15 to 2.03)	0.363	0.85 (0.19 to 3.77)	0.833
Postgraduate degree		12 (25%)	1.12 (0.29 to 4.26)	0.868	1.17 (0.25 to 5.45)	0.845	0.30 (0.07 to 1.26)	0.079	0.32 (0.07 to 1.57)	0.139
Sufficient time in residency			1.14 (0.63 to 2.03)	0.669	0.97 (0.49 to 1.93)	0.936	0.86 (0.45 to 1.63)	0.647	0.89 (0.42 to 1.87)	0.759
Sufficient time in free time			0.75 (0.40 to 1.49)	0.364	1.28 (0.62 to 2.66)	0.504	1.20 (0.60 to 2.38)	0.607	0.94 (0.43 to 2.07)	0.880
Medical school research education		33 (67.35%)	1.00 (0.29 to 3.40)	1.00	1.44 (0.32 to 6.53)	0.631	3.13 (0.79 to 12.36)	0.087	1.00 (0.21 to 4.73)	1.00
Medical school research experience		18 (36.76%)	2.37 (1.13 to 4.96)	0.022	2.39 (1.00 to 5.72)	0.051	1.98 (0.89 to 4.42)	0.095	1.35 (0.53 to 3.46)	0.528
Residency year										
PGY-1		25 (51.02%)	1.00		1.00		1.00		1.00	
PGY-2		12 (24.49%)	1.00	1.00	1.40 (0.19 to 10.06)	0.737	0.44 (0.10 to 1.94)	0.262	0.40 (0.08 to 2.11)	0.264
	PGY-3	11 (22.45%)	2.67 (0.53 to 13.32)	0.214	5.83 (0.92 to 36.96)	0.034	4.38 (0.42 to 45.36)	0.176	2.00 (0.19 to 21.29)	0.558
	PGY-4	1 (2.04%)								

### Knowledge

Residents were asked to rate their confidence with 12 discrete research tasks on a 5-point scale, with 1 being the least confident and 5 being most confident. Residents were least confident in performing multivariable regression and classic statistics (Table 2). They demonstrated greater confidence in research critical appraisal of research, such as identifying bias and confounding. Confidence levels were somewhat lower for project development tasks such as developing a study protocol, designing qualitative research, and completing an Institutional Review Board application. The notable discrepancy between confidence in research evaluation and project management indicates a possible deficiency in existing curricula, necessitation additional exploration of specific interventions.

**Table 2.** Residents were asked to rate their confidence with certain research oriented tasks using a 5-point scale ranging from least (top) to most (bottom) confident for qualitative assessment of self-reported competence.

Perform multivariable regression	(least confident)
Perform classic (non-regression) statistics	
Interpret multivariable regression	
Develop a study protocol	
Design a qualitative research study	
Interpret classic (non-regression) statistics	
Complete an Institutional Review Board Application	
Identify bias	
Identify confounding	
Interpret qualitative research	
Write a research paper	
Apply research to my day-to-day practice	(most confident)

### Resident attitudes towards research

Resident views on research were quantified using a 5-point Likert list of 7 statements (Table 3). The statement ‘I want to learn more about research’ was most highly ranked, while residents ranked ‘I feel prepared to do independent research’ lowest.

**Table 3.** Residents were asked to rate each of the following statements on a scale of 1 (strongly disagree – top) to 5 (strongly agree – bottom) for qualitative preference of attitudes and beliefs.

Statement	
I feel prepared to do independent research	(most strongly disagree)
I enjoy doing research	
Doing research is an important part of being a doctor	
Doing research will be an important part of my career	
Doing research is important for my future job	
Doing research is important for my career	
I want to learn more about research	(Most strongly agree)

### Priorities for research education

Residents were asked to rank their preference regarding research skills, study design and study population, on a 7-point Likert scale with 1 being the choice they are most interested in learning about and 7 being the choice they are least interested in. Residents expressed strongest interest in learning about observational study designs, followed by clinical trial design and writing in research. Statistics were of least interest (Table 4(a)). When residents were asked which study design they would be most interested in learning about, they ranked chart review cohort studies highest, followed by systematic reviews and clinical trials. Residents were most interested in conducting studies of rare diseases and showed less interest in studying diseases in the outpatient setting (Table 4(c)). Thematic analysis of a free text box soliciting suggestions on areas of clinical research which residents would be interested in learning about. Statistics (5), grant writing (3), early project planning and development (2), the publication process (3) and academic career planning (1) were key themes raised by residents.

**Table 4.** Residents were asked to rank their preference with regard to research skills, study design and study population, on a 7-point Likert scale with 1 being the choice they are most interested in learning about and 7 being the choice they are least interested in, for qualitative assessment of preference results are ranked in order of highest priority based on answers.

A: Residents were asked which topic is most important to their research priorities in residency.	
Observational study design	(most interested)
Clinical trial design	
Writing in research	
Practical aspects of research	
Qualitative research study design	
Classic (non-regression) statistics	
Multivariable regression	(least interested)
B: Residents were asked ‘Which of the following study designs would you like to learn more about?’	
Cohort study – chart review	(most interested)
Systematic reviews and meta-analyses	
Clinical trials	
Case control studies	
Cohort studies – prospective	
Cross sectional studies	
Community based observational research	(least interested)
C: Residents were asked ‘Which populations/demographics would you like to study during your residency?’	
People with rare diseases	(most interested)
People with common diseases	
Racial/ethnic minority groups	
Hospitalized patients	
People in low- and middle-income settings	
People being treated in specialist clinics	
People being treated in primary care settings	(least interested)

### Practices

Residents had limited awareness of certain supports, particularly support with study planning and design (18.75%), data anonymization (20.41%) and manuscript proof reading services (24.49%). Approximately half were aware of support identifying an appropriate research mentor (51.02%) and conducting a literature review (48.98%).

Most residents were interested in using routine data and national databases for research (83.67%), while less than half were able to access pre-existing databases through a supervisor (45.83%). Two thirds were interested in receiving formal statistical training (67.35%). Only a third (34.38%) always consulted with a statistician during project planning. Only a small number of people failed to define key STROBE terms during protocol development (Table 5).

**Table 5.** Resident responses to survey questions.

A: When writing a protocol, do you usually include the following definitions?				
		No	Sometimes	Yes
Exposure or case definition		4 (13.79%)	7 (24.14%)	18 (62.07%)
Primary outcome		3 (10.34%)	4 (13.79%)	22 (75.86%)
Study population		3 (10.34%)	5 (17.24%)	21 (72.41%)
Target population		3 (10.71%)	5 (17.86%)	20 (71.43%)
B: Summary of thematic analysis of barriers to research at different stages of project development				
Stage of Research	Key Barriers/Themes Identified			
All Stages:	Time constraints, statistical analysis, technical methodology issues			
Study Planning:	Identifying mentorship (3), selecting an appropriate project (4)			
Project Conduct:	Quality of old databases (1), methodology issues (1), data collection efficiency (5), failure to define all pertinent variables early			
Data Analysis:	Identifying the correct statistician (1), confounding (1), lack of statistical power (1)			
Manuscript Writing:	Motivation (2), specific journal requirements (2), data integration (2), correct language and writing skills (3)			
Peer Review Process:	Rejection (2), delays in review (4), requests for additional data (2)			

### Barriers to research

Thematic analysis was conducted for barriers to research at different stages of project development. Across most or all stages of a research project, time, statistical analysis and technical aspects relating to methodology and planning were raised. Themes unique to study planning included identified mentorship (3) and an appropriate project (4). Issues faced in conducting a project included quality of old databases (1), methodology (1) and data collection efficiency (5), particularly relating to failure to define all pertinent variables at the start of data collection. Barriers unique to data analysis included identifying the correct statistician (1), confounding (1) and lack of power (1). For manuscript writing, motivation (2), specific journal requirements (2), data integration (2) and correct language and writing skills were identified (3) as key themes. Residents frequently cited rejection (2), delays with the review process (4), requests for additional data (2) as key problems unique to the peer review process.

## Discussion

This mixed-methods survey provides valuable insights into resident knowledge, attitudes, beliefs and practices relating to research education needs. Overall, residents express a strong desire for further research training, though there was less consensus on the broader importance of research to the medical profession. The findings offer a foundation for improving research curricula by aligning educational strategies with resident preferences and addressing key barriers to research engagement.

### Resident priorities for research education

Resident preferences indicate a pragmatic approach to research engagement, favoring study designs that can be feasibly completed within residency training. Observational study designs, particularly chart review cohort studies, were ranked as the highest priority, followed by systematic reviews and clinical trials. This preference likely reflects the practical constraints of residency, where time and resource availability limit engagement in more complex research methodologies. In light of the pronounced preference for observational designs, residency programs might prioritize training in cohort studies and chart reviews, while simultaneously investigating methods to integrate interventional research for advanced trainees. Given the importance of systematic reviews in evidence synthesis, structured instruction in meta-analysis methodology may also be beneficial.

### Barriers to research productivity and mentorship

Residents identified several barriers to research, including time constraints, difficulty with study identification, and statistical challenges. Notably, mentorship was a recurring theme, with residents citing difficulty in identifying appropriate mentors and securing guidance throughout the research process. Institutions could support trainees by implementing formal mentor-matching programs to address obstacles associated with mentorship and project selection. Providing structured mentorship frameworks, including faculty development initiatives, may also improve mentor availability and engagement.

### Statistical training

Residents reported low confidence in performing multivariable regression and other statistical techniques, while expressing greater confidence in critically appraising research. This gap suggests that existing curricula may not provide adequate exposure to practical statistical applications. Despite low confidence, many residents expressed interest in formal statistical training. Encouraging early engagement with statistical resources and integrating structured statistical education focused on study design and interpretation may address this concern.

### Strengths & limitations

This study employs a semi-structured qualitative approach, incorporating both open-ended and Likert-style questions to capture nuanced perspectives. While this method provides rich insights, it is inherently subject to variation. Additionally, as this study was conducted in a well-resourced academic institution with established clinical research opportunities, findings may not generalize to all residency programs. However, the high response rate mitigates concerns regarding selection bias. Future research education needs assessment studies could helpfully include program leadership to better identify discrepancies [11].

## Conclusion

Residents demonstrate strong support for enhanced research education, particularly in observational methodologies and systematic reviews. Addressing these priorities with curricula while integrating strategies to introduce interventional research may foster greater engagement over time. Structured mentorship programs and improved access to statistical training could help mitigate common barriers to research engagement, ultimately promoting a more robust research culture within residency programs. Formal statistical training would likely be welcomed by trainees, particularly if there is a focus on interpretation of statistical testing rather than the direct deployment of these methods. Future studies should investigate the long-term effects of structured research curricula on resident career advancement and academic productivity, particularly focusing on how early training in statistics and project management might enhance research involvement.

## Supplementary Material

Supplemental_Appendix_1.docx Supplemental_Appendix_1.docx.

## Data Availability

Irrevocably anonymized survey responses are available on reasonable request. A PDF copy of the survey instrument used can be found in the supplemental appendix.

## Institutional approval

Approval was obtained for this study from the Mayo Clinic Institutional Review Board (ID 23–011040) and the Mayo Clinic Educational Research Committee (Application 23–107).
