# Supplementary material for: A mixed methods survey of research education requirements for residents in internal medicine, neurology and transitional programs
Source: Med Educ Online. 2025 Apr 24;30(1):2494579. doi: 10.1080/10872981.2025.2494579 (PMC12024497; doi:10.1080/10872981.2025.2494579)
Supplement: Supplemental_Appendix_1.docx [file ZMEO_A_2494579_SM3259.docx]

Research Curriculum Development Survey

- Required
- This form will record your name, please fill your name.

# Introduction

The Internal Medicine, Transitional Year and Neurology Residency Programs are collaborating to develop a Residency Research Curriculum. The aim is to support residents in effectively developing and conducting a clinical research project from conception to publication.

Residents have different experiences of research both during and prior to starting residency. This survey will use a Knowledge, Attitudes, Behaviors and Practices approach to explore how residents approach research, what skills they would like to develop, and what challenges they face in leading projects. By completing this survey, you will help to develop a curriculum which is designed to support residents like you.

**How we use your data:** The survey data will be collected in an un-anonymized spreadsheet, and stored in a password protected file which will not be accessible by people directly affiliated with your residency. The data will then be anonymized, and an anonymized report will be shared with your residency program leadership. We collect this un-anonymized data to be able to pair your answers here with your answers to a follow-up survey which we will send out after you have had a chance to attend the Research Curriculum.

# Participant Information

1. Which residency program are you enrolled in?

Internal Medicine

Neurology

Transitional year

Research fellow

1. After completion of your transitional year, which residency will you complete?

Radiology

Anesthesiology

Radiation Oncology

Dermatology

Diagnostic Radiology

1. What PGY year are you?

PGY-1

PGY-2

PGY-3

PGY-4

1. Aside from your MD, do you posses an additional postgraduate degree?

No

Yes

1. Which postgraduate degree do you have? (select all that apply)

MSc

MPH

PhD

PharmD

Other

1. What discipline was/were your postgraduate degree(s)

Public Health

Other

1. Are you: Male

Female

Other

1. Have you taken part in a research project before? *

Yes

No

1. Have you taken part in a research project during residency? *

Yes

No

1. Have you presented your own original research at a national or international conference before? (Select all that apply)

Yes - oral presentation

Yes - poster presentation

Yes but I was not the presenter

No

1. Have you published a first author peer-reviewed original research paper before?

No

Yes

# Knowledge

1. Did your medical school include formal teaching on clinical research methods

Yes

No

1. Did your medical school include a formal opportunity to conduct a research project?

Yes - as a lead/first author investigator

Yes - as a collaborating/non-lead investigator

No

1. To what extent do you feel you know enough about research methods to complete the following tasks?

1- Not at all

confident 2 3 4 5- very confident

Complete an IRB (ethics) application

Develop a study protocol

Interpret classic (non-regression) statistics

Perform multivariable regression

Interpret multivariable regression output

Write a research paper

Identify bias

Identify confounding

Interpret qualitative research

Apply research to my day-to-day practice

Design a qualitative research study

# Attitudes

1. Do you feel you have enough time to complete an adequate amount of research **during your working hours** and scheduled rotations to meet your professional goals in residency?

Definitely

Mostly

Somewhat

Mostly not

Definitely not

1. Do you feel you have enough time to complete an adequate amount of research **outside of working hours** to meet your professional goals in residency?

Definitely

Mostly

Somewhat

Mostly not

Definitely not

1. Please rate the following statements

| 1 strongly disagree | 2 | 3 | 4 | 5 strongly agree |
| --- | --- | --- | --- | --- |

Doing research is an important part of being a good doctor

Doing research will be an important part of my future career

I enjoy doing research

I want to learn more about research

I feel prepared to do independent research

Doing research is important for my future job

Doing research is important for my career

1. Please rank the following topics from 1 to 7 in order of relevance to your research ambitions in residency, with 1 being most important and 7 being least important.

1 2 3 4 5 6 7

Observational study design

Clinical trial design

Qualitative research study design

Classic (nonregression) statistics

Multivariable regression

Writing in research

Practical aspects of research

19. Which of the following study designs would you like to learn more about?

Please rank the following options from 1 to 7, with 1 being the study design you're most interested in learning about, and 7 being the one you're least interested in learning about.

1 2 3 4 5 6 7

Cohort studies chart review

Cohort studies prospective

Case-control studies

Cross sectional studies

Clinical trials

Systematic reviews and meta analyses

Community based observational research

20. Which populations/demographics would you like to study during your residency?

Please rank the following options from 1 to 7, with 1 being the study population you're most interested in learning about, and 7 being the one you're least interested in learning about.

1 2 3 4 5 6 7

People with rare diseases

People with

common diseases

Racial/ethnic minority groups

Hospitalized patients

People being treated in specialist clinics

People being treated in primary care settings

People in low and middle income

settings

21. What other areas of clinical research would you like to learn about during residency?

# Behaviors

22. Do you know how to access/have you previously accessed the following resources and services?

Yes No Unsure or Maybe

Statistical support for analysis

Assistance with conducting a literature review

Manuscript proof-reading services

Support with study planning

Support anonymizing a dataset

Support identifying an appropriate research mentor

IRB submission form

Educational research approval committee

1. Are you interested in using routine data and national databases for research?

Yes

No

Not sure

1. Have you been able to access a pre-existing database through a supervisor?

Yes

No

Other

1. Would you be interested in receiving formal statistical training in research?

Yes

Maybe

No

# Practices

1. Have you conducted a research project before?

Yes

No

1. When conducting a research project, do you usually write a protocol?

Yes

No

1. When writing a protocol, do you usually include the following definitions?

No Sometimes Yes

Exposure or case definition

Primary outcome

Study population

Target population

1. When conducting a research project, do you usually consult with a statistician during the planing stage?

Yes

Sometimes

No

1. What challenges have you faced in planning your research project?
2. What challenges have you faced when conducting your research project?
3. What challenges have you faced when analyzing your data?
4. What challenges did you face when writing up your project?
5. What problems have you had when going through peer review?

(leave blank if you have not been the corresponding author for a peer-reviewed paper before)

Thank you!

We appreciate you taking the time to complete this survey. Your responses have been recorded.

This content is neither created nor endorsed by Microsoft. The data you submit will be sent to the form owner.


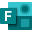


Microsoft Forms
